# Comparison of Profile Attractiveness between Class III Orthodontic Camouflage and Predictive Tracing of Orthognathic Surgery

**DOI:** 10.1155/2020/7083940

**Published:** 2020-09-07

**Authors:** Mohamad Nagi Bou Wadi, Karina Maria Salvatore Freitas, Daniel Salvatore Freitas, Rodrigo Hermont Cançado, Renata Cristina Gobbi de Oliveira, Ricardo Cesar Gobbi de Oliveira, Guilherme Janson, Fabricio Pinelli Valarelli

**Affiliations:** ^1^Department of Orthodontics, Ingá University Center UNINGÁ, Maringá, Brazil; ^2^Freitas Dentistry Institute, Bauru, Brazil; ^3^Department of Orthodontics, Bauru Dental School, University of São Paulo, Bauru, Brazil

## Abstract

**Objective:**

The aim of this study was to compare the profile attractiveness between orthodontic camouflage of the Class III malocclusion and the predictive tracing simulating orthognathic surgery evaluated by dentists and laypeople. *Settings and sample population*. The sample consisted of 21 patients (9 male; 12 female) with Class III malocclusion treated with orthodontic camouflage and Class III intermaxillary elastics. *Material and Methods*. The mean initial age of the patients was 24.38 years (SD 3.32), and the mean ANB angle was −1.91° (SD 0.83°). Patients presented skeletal Class III and normal growth patterns. Initial and final lateral cephalograms of each patient were used. The initial cephalogram was used to perform the treatment simulation of orthognathic surgery, and its silhouette was compared to the silhouette obtained from the final cephalogram after Class III orthodontic camouflage. A subjective analysis of profile attractiveness was performed by 47 laypeople and 60 dentists, with scores from 1 (less attractive) to 10 (most attractive). Mann–Whitney tests were used to compare profile attractiveness between the orthodontic treatment and the predictive tracing groups and between dentists and laypeople.

**Results:**

The predictive tracing of orthognathic surgery showed to be statistically significantly more attractive (mean score 4.57, SD 2.47) than that of the Class III camouflage orthodontic treatment (mean score 4.22, SD 2.40), with a mean numerical but significant difference of 0.35 (SD 2.01) (*P* < 0.001). Laypeople were more critical than dentists in evaluating profile attractiveness, but numerical difference between the groups was also small.

**Conclusion:**

The profile silhouette of predictive tracing simulating orthognathic surgery showed to be more attractive than that of Class III camouflage orthodontic treatment; however, differences were small but statistically significant. Laypeople showed to be more critical than dentists.

## 1. Introduction

In modern society, there is a great emphasis on attractiveness and facial beauty. The face remains one of the keys in determining human physical attractiveness [1, 2].

One of the problems that negatively interfere with facial and smile attractiveness is the presence of malocclusion. Class III malocclusion shows the greatest impairment of facial esthetics [3, 4]. This malocclusion can be orthopedically treated with maxillary expansion and reverse traction during the growth phase, but thereafter, the treatment options are camouflage orthodontic treatment or orthognathic surgery [5–7].

The importance of the Class III, besides its less prevalence among malocclusions, cannot be despised. According to the IOFTN, the Class III patients present a higher functional need for orthognathic surgery than other malocclusions [8, 9]. Most cases that are treated with orthognathic surgery in different countries present initially Class III malocclusion [8, 10–12].

The orthognathic surgery, especially in Class III malocclusion, corrects the skeletal deficiency with modification of the skeletal pattern and profile, bringing esthetics and normality aspect to that profile, teeth, occlusion, and face [13]. In some cases, even though dental malocclusion can be corrected with camouflage orthodontics compensating the skeletal discrepancy and good static and functional occlusion is achieved, an ideal facial profile esthetics may not be obtained.

Facial attractiveness and profile compromising are generally the deciding elements in treatment planning of borderline orthodontic patients who can be treated with orthodontic camouflage or orthognathic surgery [14]. In Class III patients, the facial profile and skeletal discrepancy are sometimes the main focus of the patients, and in these cases, the profile esthetics obtention should be the major goal in treatment results and long-term stability [15].

Class III malocclusion patients selected for surgical treatment are likely to have more severe skeletal discrepancy than those treated with orthodontic camouflage [16, 17]. Surgical treatment is associated with significant decompensation of the mandibular incisors [17, 18]. Class III patients treated with either camouflage or surgery treatment are likely to finish with slightly proclinated maxillary incisors [17]. Generally, surgical treatment results in greater skeletal change, involving normalization of the skeletal base relationship, reduction in chin prominence, and more favorable lips and chin contours [16, 19].

The perception and pattern of beauty and attractiveness are highly subjective and may differ among people [4, 20–22]. Attractiveness of the facial profile is controversial in the literature when comparing perceptions of laypeople and dental professionals. Some studies showed similar results among orthodontists and laypeople [23–26], while others showed divergence of their opinion [3, 22, 27, 28].

Profiles with normal mandibular anteroposterior position appear to be the most attractive, and prominent mandibles are more attractive than deficient mandibles [29]. The profile attractiveness of borderline Class III cases treated surgically or orthodontically was already compared with modified profile images masking the eyes, eyebrows, and hair, and the results showed that surgery and camouflage treatments provided similar esthetic improvement in profile attractiveness [2].

Recently, a research compared the attractiveness of the facial profile of Class III cases treated with orthodontic camouflage and orthognathic surgery [18]. The results showed that the profile attractiveness is similar in Class III patients after orthognathic surgery or orthodontic camouflage, but the surgical treatment provided more improvement in the facial profile attractiveness [18]. However, at the initial stage, cases treated with orthodontic camouflage presented more pleasant profiles than the surgically treated group, and this could have influenced the results [18].

The clinical relevance is due to the importance of knowing whether the camouflage orthodontic treatment of cases with borderline skeletal Class III malocclusion, that is, with a surgical indication, if treated with orthognathic surgery, would result in similar or greater profile attractiveness to that obtained with camouflage orthodontics.

This way, the present study aimed to compare the attractiveness of profile silhouettes in borderline skeletal Class III malocclusion patients treated with orthodontic camouflage and the predictive tracing, simulating orthognathic surgery of the same patients, evaluated by dentists and laypeople. The null hypothesis to be tested was that there is no difference in facial profile attractiveness between Class III malocclusion camouflage orthodontic treatment and the predictive tracing simulating orthognathic surgery.

## 2. Material and Methods

### 2.1. Sample

This study was approved by the Ethics in Human Research Committee of UNINGÁ University Center, and all patients signed informed consent.

Sample size calculation was based on an alpha significance level of 5% and a beta of 20% to achieve 80% of test power to detect a minimum difference of 0.9 points in the score of profile attractiveness, with a standard deviation of 1.02 [30]. This way, the sample size calculation showed the need for 21 subjects.

The sample was obtained from the records of the Orthodontic Clinic at IOPG (Post-graduation Institute of Dentistry, Bauru, Brazil). Inclusion criteria were presence of initial Class III malocclusion with at least half-cusp Class III molar relationship [31]; all permanent teeth irrupted up to the first molars; no agenesis or supernumerary teeth; no history of previous orthodontic treatment; borderline surgical/orthodontic patients, with ANB angle of −1° or less (more severe Class III malocclusion), treated with orthodontic camouflage, without extractions; and cases in which the orthodontist and the maxillofacial surgeon agreed with the need of orthognathic surgery to correct the malocclusion and the skeletal discrepancy, but the patients refused the surgery for many reasons (that we did not evaluate) and were treated with orthodontic camouflage and Class I canine and molar relationships obtained at the end of treatment. Patients with lateral cephalograms that did not show clearly the soft tissue profile were excluded from the sample. All lateral cephalograms were performed with lips in rest position, and this was confirmed with the comparison of the lateral cephalogram with the extraoral profile photographs. The diagnostic records were obtained in centric relation.

These were cases where both the orthodontist and the maxillofacial surgeon agreed with the need for orthognathic surgery to correct the malocclusion and the skeletal discrepancy, but the patients refused the surgery for many reasons (that we did not evaluate). This way, these cases were treated with the orthodontic camouflage.

The sample comprised 21 patients (9 males; 12 females) with a mean initial age of 24.38 years (SD 3.32). The mean treatment time was 2.39 years (SD 0.78). The mean ANB angle of the sample was −1.91° (SD 0.83°). Patients presented skeletal Class III and normal growth patterns (Table 1). The severity of the Class III molar relationship of the patients was as follows: 6 patients with full-cusp Class III, 7 with ¾-cusp, and 8 patients presenting half-cusp Class III molar relationship [31].

The mechanics used for Class III camouflage orthodontic treatment included fixed preadjusted appliance (Class III Biofunctional prescription, 0.022 × 0.030-inch slots, Morelli, Sorocaba, SP, and Brazil). Leveling and alignment were performed with 0.014″, 0.016″, and 0.018″ Nitinol and 0.020″ and 0.019″ x 0.025″ stainless steel archwires. All patients were treated without extractions. The main mechanics for Class III correction was the use of heavy 3/16″ Class III intermaxillary elastics. The biofunctional prescription of fixed appliances includes lingual crown torque on the maxillary anterior teeth and labial crown torque on the mandibular anterior teeth to counteract the Class III elastics [5–7].

### 2.2. Cephalometric Assessment and Simulation of Orthognathic Surgery

**Figure 1:**
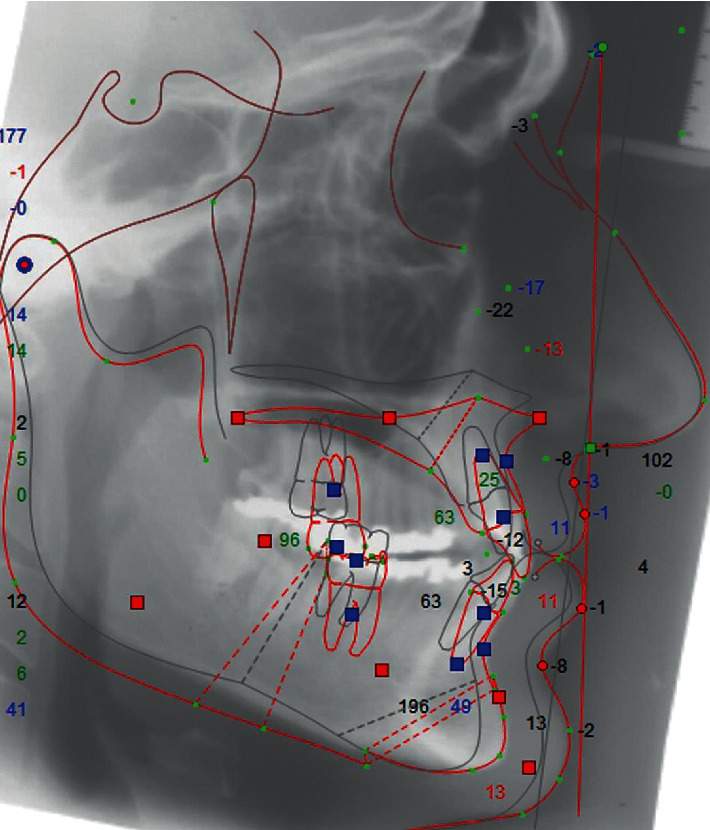
Digital predictive tracing simulating orthognathic surgery.

**Figure 2:**
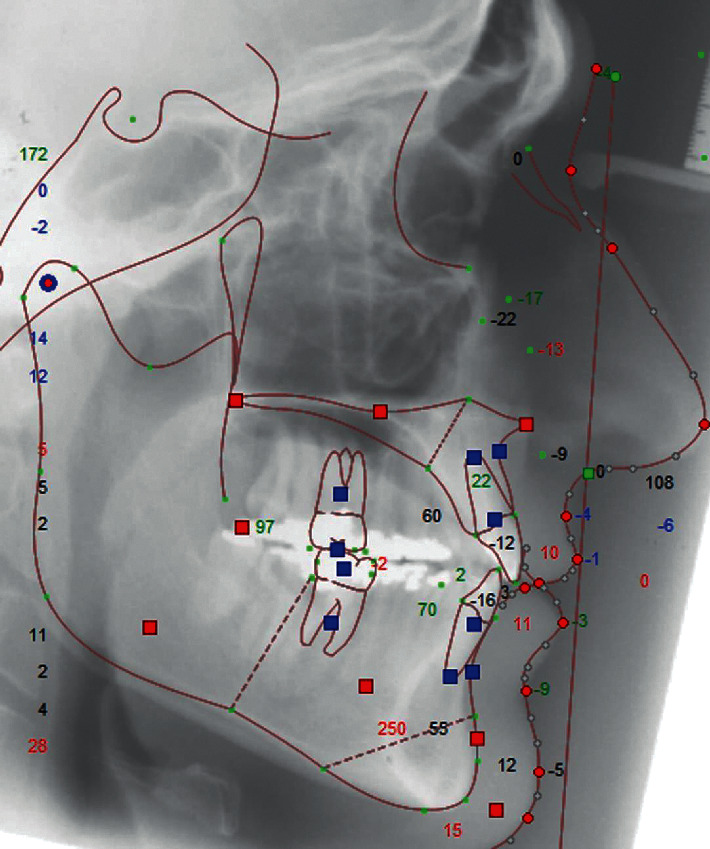
Final cephalometric tracing.

Initial and final lateral cephalograms of each patient were used. The initial cephalogram was used to perform the treatment simulation of orthognathic surgery which was compared to the final cephalogram, to evaluate the facial profile attractiveness of the Class III orthodontic camouflage (Figures 1 and 2).

The lateral cephalograms were scanned (i800 Microtek ScanMaker, Santa Fe Springs, Calif, USA) and analyzed with Dolphin Imaging and Management Solutions 11.5 software (Chatsworth, Calif, USA). The predictive tracing simulating the result of orthognathic surgery was drawn from the initial cephalogram of each patient. The cephalometric tracings and orthognathic treatment simulation were performed by a maxillofacial surgeon with experience and training (MNBW). Since the incisors were not decompensating in the initial lateral cephalogram, the decompensation of teeth was performed in the software before the surgical simulation was done. The software allows these changes in incisor's inclination to simulate the presurgical position of the incisors and then a correct simulation of the jaw's movements to be performed during orthognathic surgery.

### 2.3. Assessment of Facial Profile Attractiveness

**Figure 3:**
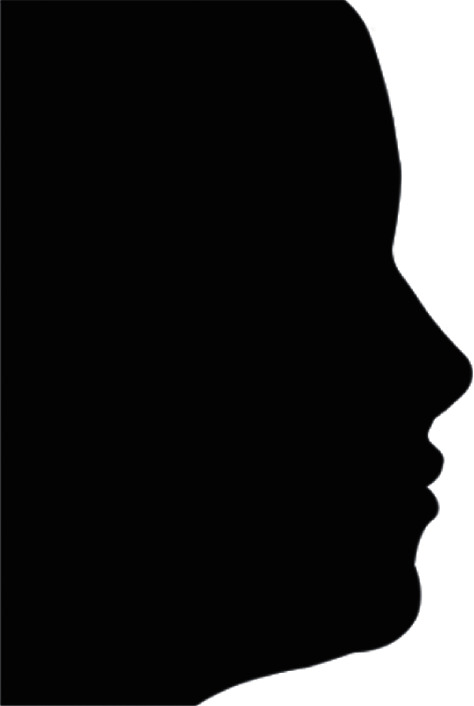
Facial profile silhouette.

Subsequently, facial profile silhouettes were constructed with CorelDRAW software (version 2017, Corel Corporation, Ottawa, Canada, Figure 3). Therefore, the profiles could be objectively evaluated without influence of facial variables such as sex, skin, hair, and eye color [26, 32].

The silhouettes were divided into two groups: Group 1: predictive tracing of orthognathic surgery and Group 2: final cephalometric tracing of Class III orthodontic camouflage. They were randomized and sequentially numbered from 1 to 42 to be evaluated.

A subjective analysis was performed by laypeople and dentists. The evaluators were invited to participate in the survey through a link sent by e-mail. An internet website powered by Google (Mountain View, CA, USA) stored the images that were evaluated by 60 dentists (27 male, 33 female, mean age 33.54 years) and 47 laypeople (22 male, 25 female, mean age 34.75 years). The group of dentists included orthodontists and orthodontic graduate students, without the formal title obtained yet, but all of them have knowledge of orthodontics. Laypeople were defined as individuals without formal education in dentistry or dental hygiene.

The profile attractiveness subjective analysis was evaluated using a numerical rating scale (NRS) [33], with scores from 1 (less attractive) to 10 (most attractive). The images were released in a sequential order. The evaluators could change the scores at any time before finalizing the survey. The answers were forwarded to the database and statistically evaluated.

### 2.4. Error Study

Reliability and precision of the methodology were verified in 10 randomly selected silhouettes, in which attractiveness was reevaluated within a month's interval by 10 randomly selected evaluators. The kappa coefficient was evaluated and showed a value of 0.92, which is considered as an excellent agreement [34].

### 2.5. Statistical Analysis

Normality of data was checked with Shapiro–Wilk tests. Since the data did not present normal distribution, nonparametric tests were used.

The Mann–Whitney test was used to compare profile attractiveness between the predictive tracing and the orthodontic camouflage groups and between dentists and laypeople.

Statistical analysis was performed with the Statistica software (STATISTICA for Windows version 7.0, Statsoft, Tulsa, Oklahoma, EUAUSA), and results were considered significant at *P* < 0.05.

## 3. Results

Patients presented skeletal Class III malocclusion indicated by the ANB and Wits appraisal and a normal equilibrated growth pattern (Table 1).

**Figure 4:**
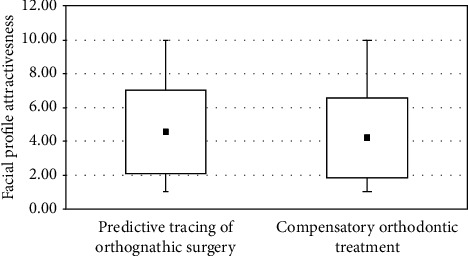
Boxplot of the score of facial profile attractiveness of predictive tracing of orthognathic surgery and orthodontic camouflage.

**Figure 5:**
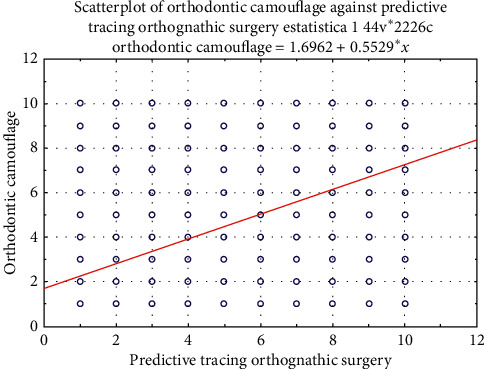
Scatterplot of the scores of facial profile attractiveness of predictive tracing of orthognathic surgery and orthodontic camouflage.

There was a statistically significant difference in the profile attractiveness between the predictive tracing of orthognathic surgery for Class III patients and their respective final cephalometric tracing after orthodontic camouflage (Table 2). The predictive tracing of orthognathic surgery performed with Dolphin software showed to be more attractive than the facial profile promoted by the orthodontic camouflage at the final stage (Table 2). However, Figures 4 and 5 show boxplots and scatterplots of this comparison, indicating the slight numerical but significant difference between the groups.

There was a statistically significant difference between the score of profile attractiveness between laypeople and dentists, for both predictive tracing of orthognathic surgery and the orthodontic camouflage, indicating that laypeople are more critical than dentists in evaluating profile attractiveness of the predictive tracing of orthognathic surgery and the orthodontic camouflage of the Class III malocclusion (Table 3).

## 4. Discussion

The rising interest in beauty and esthetics increased the search for orthodontic treatment and led orthodontists to seek treatments that would result in better facial harmony. Esthetics of the facial profile can be evaluated in different ways, but the silhouettes are a good method since it eliminates confounding factors that influence the attractiveness, such as sex, age, skin, hair, and eye color [26, 32, 35].

Borderline cases with at least half-cusp Class III malocclusion were selected in order to provide reliable results regarding the predictive tracing simulating orthognathic surgery. Predictive tracing simulating orthognathic surgery showed to be a good predictive comparative outcome [36]. The Dolphin Imaging's software is considered to be accurate within an error range of ±2 mm [37, 38].

The orthodontic camouflage was performed with the preadjusted biofunctional bracket prescription [5–7]. It has palatal crown torque in the maxillary anterior teeth and labial crown torque in the mandibular anterior teeth to produce an opposite and resistant force generated by intermaxillary Class III elastics. The result is an optimal incisor positioning with minimum side effects, correcting the Class III malocclusion, and improving the facial profile [5–7]. However, the dentoalveolar correction of a skeletal problem does not always result in the best possible outcome. This way, a comparison was performed with a profile tracing simulating orthognathic surgery in these same patients that were compensatorily treated with orthodontics only.

A numerical rating scale (NRS) [33] from 1 to 10 was used for evaluation of profile attractiveness. Other types of scales are the visual analogue scale (VAS) and the Likert scale. Although no major differences in the practical use of these modalities of scales have been found, the NRS seems to be slightly preferred, since it is easy to complete and appropriate for all groups of subjects [39, 40].

A limitation of this study is that only the facial profile silhouettes were evaluated, and both treatments, camouflage orthodontics and orthognathic surgery, change the frontal view of the patient. However, in Class III patients, the facial profile discrepancy is usually the most esthetic impairment and the main complaint of patients [9, 10]. This way, even with this limitation, we can speculate that changes in the facial profile in both treatment approaches, even if one is only a surgical simulation, will reflect in similar changes in the frontal view.

The predictive tracing simulating orthognathic surgery showed to be significantly more attractive than the profile achieved after the orthodontic camouflage of the Class III malocclusion (Table 2). This indicates that the profile of Class III patients would be more attractive if orthognathic surgery had been performed. Though there was consensus about orthognathic surgery as the first treatment option between the orthodontist and maxillofacial surgeon, surgery was indicated but declined by the patients. However, the difference of facial profile attractiveness between surgical simulation and orthodontic camouflage, besides being statistically significant, was numerically small (difference of the mean scores between the two groups was only 0.35) and probably did not imply in a clinically significant difference (Table 2 and Figure 4).

Comparing surgery and camouflage orthodontic treatment, Adamian [2] showed that both treatments provided similar esthetic improvement in profile attractiveness in borderline Class III patients. However, she used modified profile photographs showing masking only of the eyes, eyebrows, and hair and not profile silhouettes, as our study used.

Eslami et al. [41] compared Class III patients treated with camouflage or surgery and found that the Holdaway H angle and Wits appraisal was able to differentiate between these two groups of patients. Cases with a Holdaway angle greater than 10.3° and Wits appraisal greater than −5.8 mm would be treated successfully by camouflage, while those with a Holdaway angle of less than 10.3° and with Wits appraisal less than −5.8 mm can be treated surgically. Based on this model, the authors found that 81.5% of our patients were properly classified [41].

Watanabe et al. [18] compared the facial profile attractiveness of profile silhouettes between Class III cases treated with orthognathic surgery and orthodontic camouflage and found that the surgery provided a greater improvement with treatment, and the final attractiveness was similar between the groups. However, at the beginning of treatment, the surgical group presented a lesser attractive facial profile than the camouflage group, and this is a limitation of their study.

Although our results suggest that orthognathic surgery is a better treatment than orthodontic camouflage in providing an attractive and esthetic facial profile, differences were small but statistically significant. Furthermore, it has to be taken into account that we only evaluated the facial profile attractiveness, and both treatments change other aspects such as frontal facial characteristics, teeth, and occlusion, and these characteristics were not evaluated in the present study.

Laypeople showed to be more critical than dentists in evaluating the profile attractiveness of both the predictive tracing simulating orthognathic surgery and the Class III orthodontic camouflage (Table 3). However, despite the statistically significant differences between laypeople and dentists' evaluation, the numerical differences were small, with means of 0.41 and 0.31 for the predictive tracing simulating surgery and orthodontic camouflage, respectively. This corroborates the findings of previous studies, which found different scores of profile attractiveness among laypeople and clinicians [2, 22, 27, 28, 42]. According to Kerr and O'Donnell [25], orthodontic treatment was generally seen as improving facial attractiveness, but the improvement was less appreciated by laypeople.

Although laypeople were more critical, the significance in difference was very small (Table 3). Burcal et al. [43] showed that dental groups evaluating facial profiles are more accurate, but the changes were only recognized by 80% of the participant's with at least 6 mm of change between the profiles. Other studies found that clinicians rated the profile attractiveness with lesser scores than laypeople [44]. However, when orthodontists evaluate the facial profile, they consider, even unconsciously, the type of treatment and malocclusion that are being evaluated. When orthodontists have the information that they are evaluating Class III malocclusions, they seem to be less critical with some characteristics, probably because they know the difficulty in treating this type of malocclusion. In the present study, the dentists were orthodontists or orthodontic postgraduate students, and this probably justifies this difference between our results and Chung et al. [44].

Although surgical treatment may be recommended by dental specialists, self-perceptions of the facial profile are more important in the patient's decision to elect surgical correction [42]. The perception by others that the profile of patients deciding against surgery is closer to the ideal may have some influence on their decision against surgical correction of their jaw deformities [41]. The Class III severity of the patients used in the present sample was not extremely severe, and this must be considered when evaluating our results.

A limitation of this study was the use of borderline patients, with Class III malocclusions with not so severe skeletal discrepancy. Besides the digital predictive tracing simulating the orthognathic surgery performed in software is precise [37, 38], it is only an indication of the final esthetic facial result [36], being a useful tool for patient communication [45].

## 5. Conclusions

The null hypothesis was rejected because the profile silhouette of the predictive tracing showed to be more attractive than the Class III camouflage orthodontic treatment; however, differences were small but statistically significant.

Laypeople showed to be more critical than dentists.

## Figures and Tables

**Table 1:** Mean and standard deviations of the initial cephalometric measurements of the patients.

Cephalometric variables	Mean	SD
SNA (°)	80.78	1.65
SNB (°)	82.69	1.87
ANB (°)	−1.91	0.83
Wits (mm)	−4.81	2.93
FMA (°)	25.58	3.18
SN.GoGn (°)	31.54	3.02
Gonial angle (°)	123.93	4.10
*Y* axis (°)	56.48	3.98

**Table 2:** Results of comparison of the score of profile attractiveness between predictive tracing of orthognathic surgery and orthodontic camouflage (Mann-Whitey test).

Variable	Group 1, predictive tracing of orthognathic surgery *N* = 2247		Group 2, orthodontic camouflage *N* = 2247		*P*
	Median (mean)	SD (range)	Median (mean)	SD (range)	
Score of profile attractiveness	4.00 (4.57)	2.47 (1–10)	4.00 (4.22)	2.40 (1–10)	**0.000** ^*∗*^

^*∗*^Statistically significant at *P* < 0.05.

**Table 3:** Comparison of profile attractiveness scores between laypeople and dentists (Mann–Whitney test).

Score of profile attractiveness	Laypeople *N* = 987		Dentists *N* = 1260		*P*
	Median (mean)	SD (range)	Median (mean)	SD (range)	
Predictive tracing of orthognathic surgery	4.00 (4.34)	2.58 (1–10)	5.00 (4.75)	2.35 (1–10)	**0.000** ^*∗*^
Orthodontic camouflage	4.00 (4.05)	2.46 (1–10)	4.00 (4.36)	2.35 (1–10)	**0.000** ^*∗*^

^*∗*^Statistically significant at *P* < 0.05.

## Data Availability

The data used in the study are available upon request.
